# Calreticulin Mutated Essential Thrombocythemia Presenting as Acute Coronary Syndrome

**DOI:** 10.1155/2015/161764

**Published:** 2015-04-29

**Authors:** Bassel Nazha, Gwenalyn Garcia, Ruben Kandov, Marcel Odaimi

**Affiliations:** ^1^Department of Medicine, Staten Island University Hospital, Staten Island, NY 10305, USA; ^2^Department of Hematology/Oncology, Staten Island University Hospital, Staten Island, NY 10305, USA; ^3^Department of Cardiology, Staten Island University Hospital, Staten Island, NY 10305, USA

## Abstract

Essential thrombocythemia (ET) is a myeloproliferative neoplasm characterized by a clonal expansion of megakaryocytes. ET can result in both arterial and venous thrombosis. Involvement of the coronary arteries has been reported. Patients who harbor a *CALR* mutation are half as likely to suffer a thrombotic event as compared to patients with a *JAK2* mutation. We report a case of *CALR*-mutated ET whose initial disease manifestation was a non-ST segment elevation myocardial infarction.

## 1. Introduction

Essential thrombocythemia (ET) is a myeloproliferative neoplasm characterized by a clonal expansion of megakaryocytes [1]. A Janus Kinase 2 (*JAK2*) V617F activating mutation is present in 50–60% of ET cases. An additional 5–10% of patients harbor mutations in the thrombopoietin receptor gene* MPL*. Recently, mutations in the calreticulin gene (*CALR*) have been shown to occur in 67% of ET patients with nonmutated* JAK2* or* MPL* [2].

ET can result in both arterial and venous thrombosis, with the former occurring more commonly [3]. The incidence of acute coronary syndrome (ACS) in ET has been reported as 2% in a series of 891 patients and 9.4% in a series of 170 patients [4, 5].

*JAK2*-mutated patients with ET are over two times as likely to suffer a major thrombotic event as compared to* CALR*-mutated patients [6, 7]. We present a patient with previously undiagnosed* CALR*-mutated ET whose initial disease manifestation was a non-ST segment elevation myocardial infarction (NSTEMI).

## 2. Case Presentation

A 50-year-old male presented to the emergency department with a three-day history of progressive retrosternal chest pain. His medical history was significant only for acute diverticulitis one year prior to current presentation and a 3-pack year smoking history. He had no history of diabetes, hypertension, or dyslipidemia. He denied personal or family history of cardiovascular diseases, hypercoagulable states, or bleeding disorders. A review of systems was negative for headache, visual disturbances, pruritus, easy bruising, or erythromelalgia.

On presentation, the patient's blood pressure was 131/83 mm Hg and heart rate was 84 beats/minute. He was in moderate distress due to pain. Auscultation of the lungs revealed clear breath sounds. Cardiac exam was unremarkable. No hepatosplenomegaly was detected on palpation. There was no erythema or rash on skin exam.

The patient's initial CBC revealed a hemoglobin of 14.6 g/dL, hematocrit 43.9%, WBC count 13,100/mm^3^, and platelet count 1,026,000/mm^3^. During his previous emergency department visit for acute diverticulitis, his platelet count was 976,000/mm^3^. This was felt to be reactive due to acute inflammation, and he was discharged on a course of oral antibiotics. He had no subsequent outpatient follow-up.

**Figure 1:**
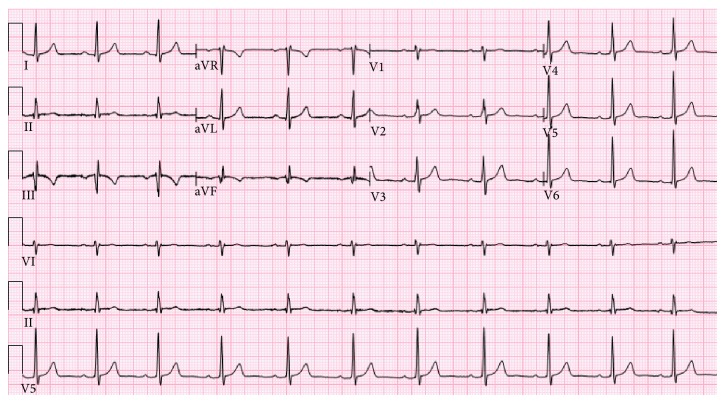
Admission electrocardiogram new showing T wave inversions in leads III and aVF, suggestive of cardiac ischemia.

An electrocardiogram showed a normal sinus rhythm with T wave inversions in leads III and aVF (Figure 1). His initial troponin I was 0.15 ng/mL. A repeat level 6 hours later increased to 2.83 ng/mL, suggesting cardiac ischemia. His LDL was 107 mg/dL, HDL 43 mg/dL, and triglycerides 98 mg/dL. His electrolytes, BUN and creatinine, and liver enzymes were within normal limits.

**Figure 2:**
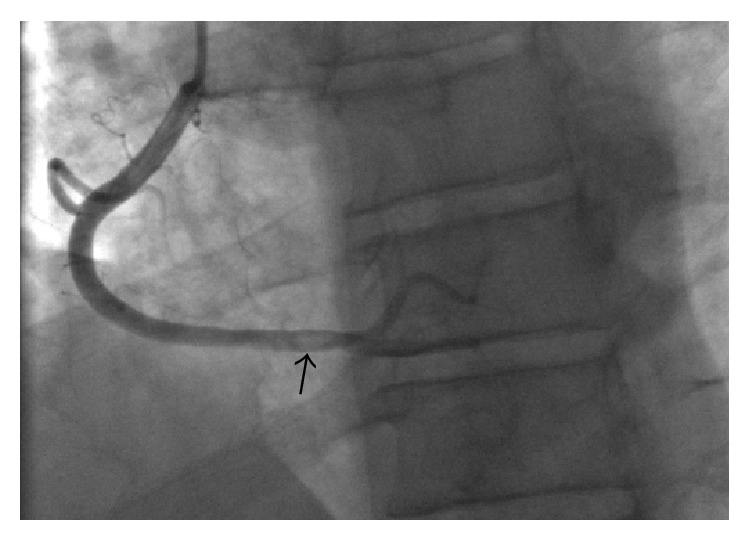
Cardiac catheterization on hospital day 1 with a thrombus (arrow) in the right coronary artery with an estimated 60% occlusion.

**Figure 3:**
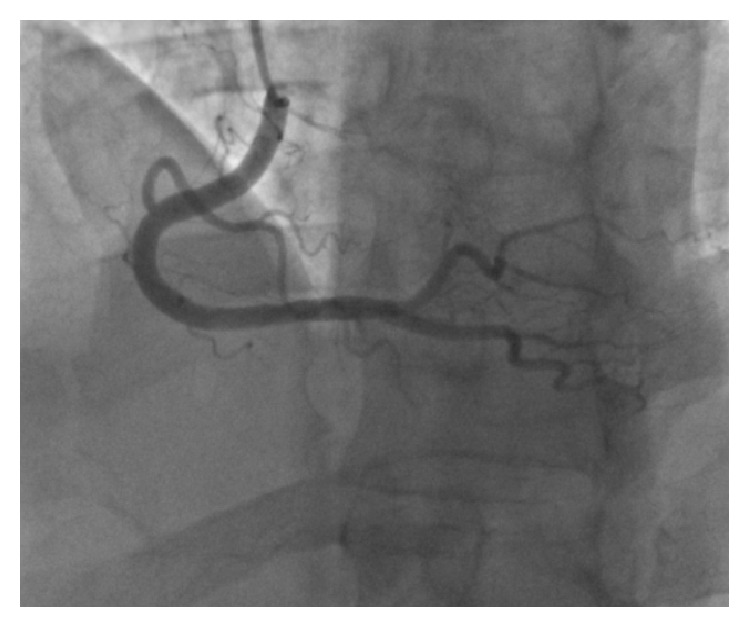
Repeat cardiac catheterization on hospital day 5 with dissolution of the right coronary artery thrombus.

The patient was transferred to the coronary care unit with a diagnosis of NSTEMI. A cardiac catheterization revealed a thrombus in the right coronary artery (RCA) with a 60% stenosis and a TIMI Grade II flow (Figure 2). No percutaneous coronary intervention (PCI) was performed due to a perceived high risk of complications, specifically distal embolization, given the high platelet count. The patient was treated with aspirin 81 mg daily, prasugrel 10 mg daily, enoxaparin 1 mg/kg every 12 hours, and hydroxyurea 1,000 mg every 12 hours. Four days later, a repeat cardiac catheterization showed normal coronaries with dissolution of the previously found RCA thrombus and no underlying atherosclerotic plaques (Figure 3).

A peripheral blood smear was significant for marked thrombocytosis without an increase in immature myeloid elements. Molecular studies were negative for* JAK2* mutations in V617F and exon 12 and positive for a* CALR* mutation type 1 (del52). Testing for* BCR/ABL* translocations was negative. Hence, a diagnosis of ET was made on the basis of the clinical presentation, consistently high platelet count, and presence of a clonal marker. 

The patient was discharged home in stable condition on hydroxyurea and low-dose aspirin. His platelet count on discharge was 821,000/mm^3^. Two months later, his platelet count normalized to 320,000/mm^3^. He continued to do well with no further thrombotic or hemorrhagic complications. A bone marrow biopsy then was hypocellular and revealed no fibrosis, dysplastic changes, or increase in blasts. Fluorescence in situ hybridization panel for myelodysplastic syndrome was also negative.

## 3. Discussion

ET is a clonal myeloproliferative disorder that causes a persistent increase in platelets. Features associated with a high risk for thrombosis include age ≥ 60 and a history of prior thrombosis. The presence of cardiovascular risk factors confers additional risk. While the degree of thrombocytosis as a risk factor is unclear, interventions that lower the count reduce the frequency of thrombotic events [3, 8].

Cytoreductive therapy with hydroxyurea has been shown to decrease the incidence of thrombotic events by 20% in high-risk patients [9]. The combination of hydroxyurea and low-dose aspirin was shown to be superior to the combination of anagrelide and low-dose aspirin in the prevention of arterial thrombosis [10]. Our patient presented with NSTEMI, putting him at high risk for a subsequent thrombotic event. Thus, we opted for treatment with hydroxyurea in addition to dual antiplatelet therapy.

In a retrospective clinical series of 1,144 ET patients, Montanaro et al. report a 1.4% annual rate of thrombotic events, with coronary involvement being the third in terms of frequency [11]. Coronary thrombi are platelet-rich and may occur at sites with no underlying atherosclerotic plaques [12, 13]. In the presented case, the repeat cardiac catheterization that was normal is consistent with a de novo thrombus formation as a primary cause for the patient's NSTEMI. Smoking is known to increase the risk of arterial complications in ET [9]; our patient's three-pack year history may have contributed to the event.

Insufficient data exist to clearly guide the treatment of ACS in ET. PCI appears to have poor outcomes for thrombotic lesions since distal embolization of thrombotic material often occurs [14]. This was the reason why our team did not attempt PCI during cardiac catheterization. Nonetheless, successful cases of PCI for ACS in ET are reported [15, 16]. The combination of hydroxyurea and aspirin is an accepted treatment strategy for patients at high risk of thrombosis [3]. Anagrelide, due to its increased risk of arterial thrombosis when compared to hydroxyurea [10], as well as rare but serious cardiovascular side effects including ACS, limits its use in patients with ET and ACS [17, 18].

Recently, mutations in exon 9 of the* CALR* gene have been found in 15–24% of the ET population.* CALR* mutations are mutually exclusive with* JAK2* and* MPL* mutations. Calreticulin is an endoplasmic reticulum protein that regulates calcium homeostasis and quality control of proteins. The exact mechanism by which* CALR* mutations produce the ET phenotype is at present unclear [6, 7].

*CALR*-mutated patients with ET have been found to have higher platelet counts, lower WBC counts, and lower hemoglobin values as compared to* JAK2*-mutated patients. Their clinical course is characterized by a thrombotic risk about half that of* JAK2*-mutated patients [6, 7]. To our knowledge, this is the first reported case of ACS in a patient with documented* CALR*-mutated ET.

Our case adds to the increasing body of literature documenting ACS in the setting of ET and normal coronary arteries. Although* CALR*-mutated patients are considered to have a more favorable clinical course as compared to* JAK2*-mutated patients, they remain at risk for major thrombotic events. Further studies aiming at identifying distinct clinical features of this newly discovered subset of patients with ET are warranted.
